# Fourier ptychographic microscopy with sparse representation

**DOI:** 10.1038/s41598-017-09090-8

**Published:** 2017-08-17

**Authors:** Yongbing Zhang, Pengming Song, Jian Zhang, Qionghai Dai

**Affiliations:** 1 Shenzhen Key Lab of Broadband Network and Multimedia, Graduate School at Shenzhen, Tsinghua University, Shenzhen, 518055 China; 2 School of Electronic and Computer Engineering, Peking University Shenzhen Graduate School, Shenzhen, 518055 China; 3 Department of Automation, Tsinghua University, Beijing, 100084 China

## Abstract

Fourier ptychographic microscopy (FPM) is a novel computational microscopy technique that provides intensity images with both wide field-of-view and high-resolution. By combining ideas from synthetic aperture and phase retrieval, FPM iteratively stitches together a number of variably illuminated, low-resolution intensity images in Fourier space to reconstruct a high-resolution complex sample image. Although FPM is able to bypass the space-bandwidth product (SBP) limit of the optical system, it is vulnerable to the various capturing noises and the reconstruction is easy to trap into the local optimum. To efficiently depress the noise and improve the performance of reconstructed high-resolution image, a FPM with sparse representation is proposed in this paper. The cost function of the reconstruction is formulated as a regularized optimization problem, where the data fidelity is constructed based on a maximum likelihood theory, and the regulation term is expressed as a small number of nonzero elements over an appropriate basis for both amplitude and phase of the reconstructed image. The Nash equilibrium is employed to obtain the approximated solution. We validate the proposed method with both simulated and real experimental data. The results show that the proposed method achieves state-of-the-art performance in comparison with other approaches.

## Introduction

Fourier ptychographic microscopy^1^ (FPM) is a novel computational imaging method which is capable of providing a scalable space-bandwidth product (SBP) for most existing microscopes. In this method, illumination angles are scanned sequentially with a programmable LED array source, while taking a low-resolution (LR) image at each angle. Assuming that illuminating a thin sample by an oblique plane wave is equivalent to shifting the center of the sample’s spectrum in the Fourier domain, each off-axis LED shifts different amounts of high spatial frequency information, diffracted from the sample, into the acceptance angle of an objective lens^2^. By capturing a stack of LR images that cover a wide region of Fourier domain and stitching them together coherently, one can achieve spatial resolution beyond the objective’s diffraction limit, corresponding to the sum of illumination and objective numerical aperture (NA)^3^.

In practice, however, the reconstruction of FPM is sensitive to the input noise. Recently, multiple algorithms have been proposed to address the noise. Generally, these algorithms utilize maximum likelihood theory which provides a flexible framework for formulating the FPM optimization problem with various noise models. If the measured images suffer only from white Gaussian noise, the negative log-likelihood function would be reduced to least squares formulation^3^. In the case of Poisson shot noise, Bian *et al*.^4^ proposed a FPM reconstruction method termed as truncated Poisson Wirtinger Fourier ptychographic (TPWFP) reconstruction. This method incorporated Poisson maximum likelihood objective function and truncated Wirtinger gradient^5^ together into a gradient-descent optimization framework. Based on the cost functions of existing FPM algorithms, Yeh *et al*.^3^ tested amplitude-based algorithms and intensity-based algorithms. The results demonstrated that amplitude-based Newton’s method gives a better reconstruction but needs much more running time for the reconstruction. Recently, Zhang *et al*.^6^ proposed a generalized Anscombe transform based approximation model (GATFP) for FPM reconstruction, which could efficiently reduce the noises. However, all the mentioned methods neglect the sparse priority of the reconstructed image, and the performance is not as good as expected.

Based on the GATFP, we propose a FPM reconstruction method termed Fourier ptychographic with sparse representation (FPSR), which brings together sparse representation^7, 8^ and Nash equilibrium^9^ to improve the performance of FPM reconstruction. Sparse representation is based on the approximation that one image could be expressed as a combination of few atomic functions taken from a certain dictionary. However, to successfully employ sparse representation, we typically have to address the additional problem of how to correctly choose the dictionary. Clearly, a proper dictionary should be an overcomplete system with a number of elements essentially larger than the dimensionality of the approximated images. Numerous pioneering works have been proposed to address the overcomplete dictionary. Christensen^10^ presented the general theory for frames and Riesz bases, where frames are generalization of the concept of basis to the case when the atomic functions are linearly dependent and form an overcomplete system. Recently, Danielyan *et al*.^11^ constructed analysis and synthesis frames, formalizing the Block Matching 3-D (BM3D)^12^ image modeling and used these frames to develop novel iterative deblurring algorithms. The other important technique incorporated in the proposed FPSR is Nash equilibrium^9, 13^ presented by Nash. This technique was originally developed for game theory, aiming at solving multiple objective optimization problems originating from game theory. In Nash’s work^13^, Non-Cooperative Games were defined as mixed strategy Nash equilibrium for any game with a finite set of actions and it was proved that at least one (mixed strategy) Nash equilibrium must exist in such a game. The Nash equilibrium is a solution concept of a game involving two or more players, in which each player is assumed to know the equilibrium strategies of the other players, and no player has anything to gain by changing only his own strategy unilaterally^14, 15^. If each player has chosen a strategy and no player can benefit by changing his or her strategy while the other players keep theirs unchanged, then the current set of strategy choices and the corresponding payoffs constitute a Nash equilibrium^15^. This strategy is very closely related to the nature of the proposed FPSR model where multiple objective functions should be considered.

In this paper, we assume that the intensity capturing process is subject to various signal-dependent errors, and the detected photons follow mixed Poisson-Gaussian noise. We also introduce the sparsity hypothesis^16, 17^ for both the amplitude and phase as the constraint in the reconstruction to improve the accuracy of reconstruction. To illustrate the effectiveness of the proposed method, we compare our method with three state-of-the-art algorithms, including Newton method, TPWFP, GATFP on both simulated and real data. Besides, we also compare the method incorporating sparse representation in the Newton method, termed as NSR for short. In NSR, the Newton method does not involve the background subtraction step. Our results show that the proposed method reconstructs more accurate results compared with other state-of-the-art algorithms.

## Methods

As detailed described in ref. 1, a typical FPM consists of an LED array, a light microscope with a low NA objective lens, and a monochromatic CCD camera. By sequentially turning on the LED elements on the array, the sample is illuminated from different angles, which correspond to a shift proportional to the angle of the illumination in Fourier space. Consequently, the estimated intensity **I**_*n*_ at the detector imaging plane can be generated as:

1 $${{\bf{I}}}_{n}=|{ {\mathcal F} }^{-1}\{P({\bf{k}})S({\bf{k}}-{{\bf{k}}}_{n})\}{|}^{2},$$

where ${ {\mathcal F} }^{-1}$ is the 2D inverse Fourier transform, **k**_*n*_ is the spatial frequency corresponding to the *n* ^*th*^ LED, *P*(·) represents the pupil function of the system and *S*(·) represents the Fourier spectrum of the object. For multivariate problems such as Eq. (1), it is convenient to reformulate the problem using linear algebra. Similar to ref. 18, the relation between the high-resolution (HR) reconstruction and the LR observations corresponds to two sequential linear operation:

- down-sampling caused by the object aperture
- inverse Fourier transform to the LR spectrum bands caused by the microscope imaging system

We treat these two operations as a whole and use **A** to represent this combinational sampling process. The image formation model (1) can be finally vectorized as:

2 $${{\bf{I}}}_{n}=|{ {\mathcal F} }^{-1}\{P({\bf{k}})S({\bf{k}}-{{\bf{k}}}_{n})\}{|}^{2}=|{{\bf{A}}}_{n}S{|}^{2}\mathrm{.}$$

Many methods have been developed to obtain the optimal *S*(·) utilizing the following optimization function:

3 $$\mathop{{\rm{\min }}}\limits_{S}\sum _{n}\sum _{r}|\sqrt{{{\bf{I}}}_{n}}-|{ {\mathcal F} }^{-1}\{P({\bf{k}})S({\bf{k}}-{{\bf{k}}}_{n})\}{||}^{2}=\mathop{{\rm{\min }}}\limits_{S}\sum _{n}\sum _{r}|\sqrt{{{\bf{I}}}_{n}}-|{{\bf{A}}}_{n}S{||}^{2},$$

where **r** = (*x*, *y*) is the real-space coordinate vector. Apparently, most of these methods neglect the sparse priority of reconstructed images. Motivated by this, a FPM with sparse representation was proposed in this paper. We formulate the reconstruction as the following minimization problem:

4 $$\begin{array}{c}\hat{s}=arg\mathop{{\rm{\min }}}\limits_{s}{\mathscr{D}}(S)+{\tau }_{a}{R}_{a}({s}_{a})+{\tau }_{\phi }{R}_{\phi }({s}_{\phi }),\\ s\mathrm{.}t\mathrm{.}\quad \quad S= {\mathcal F} (s),\\ s\mathrm{.}t\mathrm{.}\quad \quad s={s}_{a}\circ exp(j{s}_{\phi }),\end{array}$$

where ${\mathscr{D}}(S)$ is the data-fidelity term, *R*_*a*_ and *R*_*φ*_ are the regulation terms, *s*_*a*_ represents the amplitude of *s*, *s*_φ_ represents the phase angle of *s*, ${\tau }_{a} > \mathrm{0,}\,{\tau }_{\phi } > 0$ are the regulation parameters, respectively. To efficiently depress the noise and obtain better performance, we provide a data-fidelity based on noisy observation modelling and a regulation term based on sparse representation, which will be described in the following.

### Data-fidelity based on noisy observation modelling

Amongst the existing noise models for the FPM reconstruction, almost all of the methods are based on the Gaussian or Poisson noise. However, the capturing process of many imaging devices is subject to various signal-dependent errors, and a standard way to model these errors is to consider them as Poisson-Gaussian noise^19^. Recently, a mixed Poisson-Gaussian model^6^ was proposed for FPM reconstruction and obtained a satisfactory performance under different types of noise. This paper describes an extension of the mixed model to properly handle different types of noises and improve the robustness of the reconstruction. Now we express a brief overview of the mixed Poisson-Gaussian model and introduce the data-fidelity term. Given a noisy image **y**_*n*_ with the form

5 $$\begin{array}{l}{{\bf{y}}}_{n}={\tilde{{\bf{I}}}}_{n}+{{\bf{w}}}_{n},\quad n=\mathrm{1,}\ldots ,N,\end{array}$$

where subscript *n* is the *n* ^*th*^ image, **I**_*n*_ is the true image, ${\tilde{{\bf{I}}}}_{n}$ represents the image related to **I**_*n*_ and ${\tilde{{\bf{I}}}}_{n}\sim Poisson({{\bf{I}}}_{n})$. **w**_*n*_ is independent identically distributed zero-mean Gaussin noise component with variance *σ* ^2^, ${{\bf{w}}}_{n}\sim {\mathscr{N}}\mathrm{(0,}{\sigma }^{2})$. Specifically, we could easily obtain the likelihhood function as ref. 20:

6 $$\begin{array}{l}p({{\bf{y}}}_{n}|{{\bf{I}}}_{n})=\prod _{i=1}^{{m}^{2}}(\sum _{j=1}^{+\infty }\frac{{e}^{-{[{{\bf{I}}}_{n}]}_{i}}{[{{\bf{I}}}_{n}]}_{i}^{j}}{j!}\frac{{e}^{-\frac{1}{2{\sigma }^{2}}{({[{y}_{n}]}_{i}-j)}^{2}}}{\sqrt{2\pi {\sigma }^{2}}}),\end{array}$$

where, for every *i* ∈ 1, …, *m* ^2^, [**I**_*n*_]_*i*_ and [**y**_*n*_]_*i*_ denote each pixel in **I**_*n*_ and **y**_*n*_. However, it is not appropriate to use this function directly as the noise model, since the infinite Gaussian mixture distribution in Eq. (6) makes it difficult to obtain the gradient of the function. Furthermore, the complicated gradient will inevitably increase the processing time. Fortunately, based on the inspiration of variance stabilizing transform, the Generalized Anscombe Transform (GAT)^21, 22^ is proposed to approximate the exact likelihood. Using the GAT approximation, the likelihood of ${\tilde{{\bf{y}}}}_{n}$ with components ${\tilde{{\bf{y}}}}_{n}=2\sqrt{{{\bf{y}}}_{n}+\frac{3}{8}+{\sigma }^{2}}$ is approximately given by refs 6 and 23:

7 $$\begin{array}{l}p({{\bf{y}}}_{n}|{{\bf{I}}}_{n})=\prod _{i=1}^{{m}^{2}}\frac{1}{\sqrt{2\pi }}exp(-\frac{1}{2}{({[{\tilde{{\bf{y}}}}_{n}]}_{i}-2\sqrt{|{[{{\bf{A}}}_{n}S]}_{i}{|}^{2}+\frac{3}{8}+{\sigma }^{2}})}^{2})\mathrm{.}\end{array}$$

The negative log-likelihood function, assuming that the measurements are independent from each other, is a function of the parameters *S*, given the observed data **y**_*n*_:

8 $$\begin{array}{rcl}min\,f(S) & = & -log(\prod _{n\mathrm{=1}}^{N}p(\tilde{{{\bf{y}}}_{n}}))\\  & = & -log(\prod _{n=1}^{N}\prod _{i=1}^{{m}^{2}}\frac{1}{\sqrt{2\pi }}exp(-\frac{1}{2}{({[\tilde{{{\bf{y}}}_{n}}]}_{i}-2\sqrt{|{[{{\bf{A}}}_{n}S]}_{i}{|}^{2}+\frac{3}{8}+{\sigma }^{2}})}^{2}))\\  & = & -\frac{1}{2}\sum _{n=1}^{N}\sum _{i=1}^{{m}^{2}}(-log2\pi -{[\tilde{{{\bf{y}}}_{n}}]}_{i}^{2}+4{[\tilde{{{\bf{y}}}_{n}}]}_{i}\sqrt{|{[{{\bf{A}}}_{n}S]}_{i}{|}^{2}+\frac{3}{8}+{\sigma }^{2}}\\  &  & -4(|{[{{\bf{A}}}_{n}S]}_{i}{|}^{2}+\frac{3}{8}+{\sigma }^{2}))\mathrm{.}\end{array}$$

The first two terms are constant offsets that can be either ignored or computed prior to the reconstruction. Thus we can obtain the data-fidelity for the proposed reconstruction as^6^:

9 $$\begin{array}{l}{\mathscr{D}}(S)=-\frac{1}{2}\sum _{n=1}^{N}\sum _{i=1}^{{m}^{2}}(4{[\tilde{{{\bf{y}}}_{n}}]}_{i}\sqrt{|{[{{\bf{A}}}_{n}S]}_{i}{|}^{2}+\frac{3}{8}+{\sigma }^{2}}-4(|{[{{\bf{A}}}_{n}S]}_{i}{|}^{2}+\frac{3}{8}+{\sigma }^{2}))\mathrm{.}\end{array}$$

### Regulation term based on sparse representation

To efficiently express the sparse coding for complex value^24^, we employ the separate sparse real-valued representations for amplitude and absolute phase image. The sparse representation is formulated as the following matrix operation:

10 $${s}_{a}=abs\,(s)={{\rm{\Psi }}}_{a}{{\rm{\Theta }}}_{a},\quad \quad \quad \quad {s}_{\varphi }=angle\,(s)={{\rm{\Psi }}}_{\phi }{{\rm{\Theta }}}_{\phi },$$

11 $${{\rm{\Theta }}}_{a}={{\rm{\Phi }}}_{a}\cdot abs(s)={{\rm{\Phi }}}_{a}\cdot {s}_{a},\quad {{\rm{\Theta }}}_{\varphi }={{\rm{\Phi }}}_{\phi }\cdot angle\,(s)={{\rm{\Phi }}}_{\phi }\cdot {s}_{\phi },$$

where Θ_*a*_ and Θ_*ϕ*_ are vectors of the amplitude and phase spectra. The modulus and angle operations applied to vetors in Eqs (10) and (11) are elementwise. Thus *s*_*a*_ and *s*_*φ*_ are the vectors of amplitude and phase values. In (10), the amplitude *s*_*a*_ and phase *s*_*φ*_ are synthesized from the amplitude and phase spectra Θ_*a*_ and Θ_*ϕ*_. On the other hand, the analysis in Eq. (11) gives the spectra for amplitude and phase of the sample *s*. In Eqs (10–11) the synthesis and analysis matrices are denoted as Ψ_*a*_, Ψ_*φ*_ and Φ_*a*_, Φ_*φ*_, respectively. Following the sparsity rationale we assume that amplitude and phase spectra, Θ_*a*_ and Θ_*φ*_, respectively, are sparse; i.e., most elements thereof are zero. In order to quantify the level of sparsity of Θ_*a*_ and Θ_*φ*_, i.e., their number of non-zero (active) elements, we use the pesudo *l*_0_-norm ||·||_0_ defined as the number of non-zero elememts of the vector-argument. Therefore, we will design estimation criteria promoting low values of ||Θ_*a*_||_0_ and ||Θ_*φ*_||_0_^17^.

Two principally different variational formulations classified as the analysis and synthesis approaches can be viewed for sparse modelling. In the synthesis approach, the relation between the signal and spectrum variables are given by the synthesis in Eq. (10), while in the analysis approach these relations are given by the analysis in Eq. (11)^17^.

Based on Eq. (4), the variational setup in the synthesis approach is of form

12 $$\begin{array}{c}\mathop{max}\limits_{{{\rm{\Theta }}}_{a},{{\rm{\Theta }}}_{\phi }}\,-\frac{1}{2}\sum _{n=1}^{N}\sum _{i=1}^{{m}^{2}}(4{[\tilde{{{\bf{y}}}_{n}}]}_{i}\sqrt{|{[{{\bf{A}}}_{n}S]}_{i}{|}^{2}+\frac{3}{8}+{\sigma }^{2}}\\ \quad \quad \,\,-4(|{[{{\bf{A}}}_{n}S]}_{i}{|}^{2}+\frac{3}{8}+{\sigma }^{2}))+{\tau }_{a}{||{\rm{\Theta }}}_{a}{||}_{0}+{\tau }_{\phi }{||{\rm{\Theta }}}_{\phi }{||}_{0},\\ s\mathrm{.}t\mathrm{.}\quad \quad { {\mathcal F} }^{-1}(S)=({{\rm{\Psi }}}_{a}{{\rm{\Theta }}}_{a})\circ exp(j{{\rm{\Psi }}}_{\phi }{{\rm{\Theta }}}_{\phi }\mathrm{).}\end{array}$$

The first summand in Eq. (12) is the negative log-likelihood function corresponding to the approximated version of mixed Poisson-Gaussian distribution in Eq. (7). The pseudo *l*_0_-norms with the coefficients *τ*_*a*_ and *τ*_*φ*_ are included in order to enable the sparsity of the amplitude and phase^17^. It is clear that the synthesis setup leads to quite complex optimization problem. Correspondingly, in the analysis approach the variational setup has similar form and the same problem exists. Similar to ref. 17, a different Nash equilibrium approach is used to tackle this problem. The constrained optimization with a single criterion function, as in Eq. (12), is replaced by a search for the Nash equilibrium balancing two criteria. Demonstrations of this approach for the synthesis-analysis sparse inverse imaging can be seen in ref. 11, where it is devised for linear real-valued observation modeling. According to the above analysis, we introduce two criteria for formalization of the algorithm design:

13 $${ {\mathcal L} }_{1}(S)=-\frac{1}{2}\sum _{n=1}^{N}\sum _{i=1}^{{m}^{2}}(4{[\tilde{{{\bf{y}}}_{n}}]}_{i}\sqrt{|{[{{\bf{A}}}_{n}S]}_{i}{|}^{2}+\frac{3}{8}+{\sigma }^{2}}-4(|{[{{\bf{A}}}_{n}S]}_{i}{|}^{2}+\frac{3}{8}+{\sigma }^{2})),$$

14 $${ {\mathcal L} }_{2}({{\rm{\Theta }}}_{a},{{\rm{\Theta }}}_{\phi },{s}_{a},{s}_{\phi })={\tau }_{a}\parallel {{\rm{\Theta }}}_{a}{\parallel }_{0}+{\tau }_{\phi }\parallel {{\rm{\Theta }}}_{\phi }{\parallel }_{0}+\frac{1}{2}\parallel {{\rm{\Theta }}}_{a}-{{\rm{\Phi }}}_{a}\cdot {s}_{a}{\parallel }_{2}^{2}+\frac{1}{2}\parallel {{\rm{\Theta }}}_{\phi }-{{\rm{\Phi }}}_{\phi }\cdot {s}_{\phi }{\parallel }_{2}^{2},$$

where $S= {\mathcal F} (s)$ and ||·||_2_ stands for the Euclidean norm. The criterion (13) is identical to (9). The sparsity is enabled by the criterion (14).

Minimization of ${ {\mathcal L} }_{1}(S)$ requires the calculation of gradient. Inspired by previous works^4, 5, 25^, we can obtain the gradient of ${ {\mathcal L} }_{1}(S)$ as:

15 $$\begin{array}{rcl}\nabla { {\mathcal L} }_{1}(S) & = & \frac{d{ {\mathcal L} }_{1}(S)}{d{S}^{\ast }}\\  & = & \frac{d\{-2\sum _{n=1}^{N}\sum _{i=1}^{{m}^{2}}({[\tilde{{{\bf{y}}}_{n}}]}_{i}\sqrt{|{[{{\bf{A}}}_{n}S]}_{i}{|}^{2}+\frac{3}{8}+{\sigma }^{2}}-(|{[{{\bf{A}}}_{n}S]}_{i}{|}^{2}+\frac{3}{8}+{\sigma }^{2}))\}}{d{S}^{\ast }}\\  & = & -2\sum _{n=1}^{N}\sum _{i=1}^{{m}^{2}}\frac{d\{({[\tilde{{{\bf{y}}}_{n}}]}_{i}\sqrt{|{[{{\bf{A}}}_{n}S]}_{i}{|}^{2}+\frac{3}{8}+{\sigma }^{2}}-(|{[{{\bf{A}}}_{n}S]}_{i}{|}^{2}+\frac{3}{8}+{\sigma }^{2}))\}}{d{S}^{\ast }}\\  & = & -2\sum _{n=1}^{N}\sum _{i=1}^{{m}^{2}}\{\frac{1}{2}{[\tilde{{{\bf{y}}}_{n}}]}_{i}\frac{{[{{\bf{A}}}_{n}]}^{H}{[{{\bf{A}}}_{n}S]}_{i}}{\sqrt{|{[{{\bf{A}}}_{n}S]}_{i}{|}^{2}+\frac{3}{8}+{\sigma }^{2}}}-{[{{\bf{A}}}_{n}]}^{H}{[{{\bf{A}}}_{n}S]}_{i}\},\end{array}$$

where [**A**_*n*_]^*H*^ denotes the conjugate transpose of [**A**_*n*_].

In addition, the hard and soft thresholding operators^26^ are employed, and the solutions of ${ {\mathcal L} }_{2}({{\rm{\Theta }}}_{a},{{\rm{\Theta }}}_{\phi },{s}_{a},{s}_{\phi })$ with respect to Θ_*a*_ and Θ_*φ*_ are expressed as

16 $${\hat{{\rm{\Theta }}}}_{a}=({{\rm{\Phi }}}_{a}\cdot {s}_{a})\circ {\bf{1}}[|{{\rm{\Phi }}}_{a}\cdot {s}_{a}|\ge \sqrt{2{\tau }_{a}}],$$

17 $${\hat{{\rm{\Theta }}}}_{\varphi }=({{\rm{\Phi }}}_{\phi }\cdot {s}_{\phi })\circ {\bf{1}}[|{{\rm{\Phi }}}_{\phi }\cdot {s}_{\phi }|\ge \sqrt{2{\tau }_{\phi }}],$$

where $\sqrt{2{\tau }_{a}}$ and $\sqrt{2{\tau }_{\phi }}$ are the thresholds for the amplitude and phase, respectively. In this paper, $\sqrt{2{\tau }_{a}}=1.4$; $\sqrt{2{\tau }_{\phi }}=1.4$. Here, **1**[*u*] is an elementwise vector function, **1**[*u*] = 1 if *u* ≥ 0 and **1**[*u*] = 0 if *u* < 0. The value of Θ_*a*_ and Θ_*φ*_, which are smaller than the corresponding thresholds, are set to zero.

**Algorithm 1:**
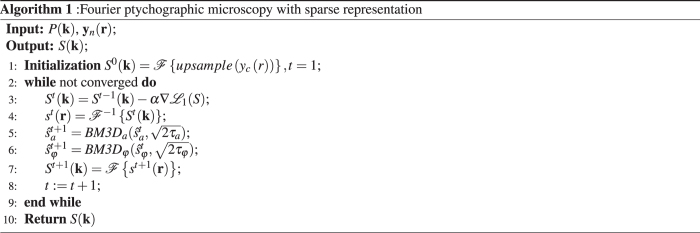
Fourier ptychographic microscopy with sparse representation.

According to the idea of the Nash equilibrium balancing multiple penalty function, the proposed algorithm is composed of alternating optimization steps performed for ${ {\mathcal L} }_{1}$ and ${ {\mathcal L} }_{2}$ ^17, 27^. The alternating optimizaiton steps could be:

18 $${\hat{S}}^{t}=arg\mathop{{\rm{\min }}}\limits_{S}{ {\mathcal L} }_{1}(S),$$

19 $$({\hat{{\rm{\Theta }}}}_{a}^{t},{\hat{{\rm{\Theta }}}}_{\phi }^{t})=arg\mathop{{\rm{\min }}}\limits_{{{\rm{\Theta }}}_{a},{{\rm{\Theta }}}_{\phi }}{ {\mathcal L} }_{2}({{\rm{\Theta }}}_{a},{{\rm{\Theta }}}_{\phi },{s}_{a},{s}_{\phi }),$$

20 $${\hat{s}}_{a}^{t+1}={{\rm{\Psi }}}_{a}{\hat{{\rm{\Theta }}}}_{a}^{t},\quad {\hat{s}}_{\phi }^{t+1}={{\rm{\Psi }}}_{\phi }{\hat{{\rm{\Theta }}}}_{\phi }^{t},$$

21 $${\hat{S}}^{t+1}= {\mathcal F} \{[{\hat{s}}_{a}^{t+1}\circ exp(j\cdot {\hat{s}}_{\phi }^{t+1})]\}\mathrm{.}$$

To enable sparse approximations, the dictionary should be rich enough to grasp all variety of images. In our algorithm, we use BM3D frames for the analysis and synthesis operations. The frame is a generalization of the concept of basis to the case when the dictionary forms an overcomplete system. The BM3D filter can be split into three steps^11^.

- *Analysis*. Similar image blocks are collected in groups in order to obtain highly correlated data. Blocks in each group are stacked together to form a 3-D data array, which is decorrelated using an invertible 3-D transform.
- *Processing*. Obtained 3-D group, which are filtered by hard-thresholding.
- *Synthesis*. The filtered spectra are inverted, providing estimates for each block in the group. These blockwise estimates are returned to their original position, and the final image reconstruction is calculated as a weighted average of all of the obtained blockwise estimates.

It follows from Eq. (20) that the steps Eqs (19) and (20) including the *Analysis* step defining the analysis Θ and synthesis Ψ frames can be combined in a single algorithm. In the remainder of the manuscript, we use the notation *BM3D* for this algorithm. Note the standard BM3D algorithm^12^ is composed of two successive steps: thresholding and Wiener filtering. However, in this paper, *BM3D* consists of only the first thresholding step. Using the *BM3D* algorithm for implementation of the Eqs (19) and (20) we obtain:

22 $${\hat{s}}_{a}^{t+1}=BM3{D}_{a}({\hat{s}}_{a}^{t},\sqrt{2{\tau }_{a}}),$$

23 $${\hat{s}}_{\phi }^{t+1}=BM3{D}_{\phi }({\hat{s}}_{\phi }^{t},\sqrt{2{\tau }_{\phi }})\mathrm{.}$$

Combining the solutions for Eq.(18) and Eqs (22–23), we obtain the FPSR algorithm shown in Algorithm 1. *y*_*c*_ in Algorithm 1 represents the image captured with central LED.

## Results

In this section, we conduct a series of experiments on both simulated and real captured data.

### Quantitative metric and parameter settings

Besides the visual results, we also introduce the relative error (RE)^4–6^ to quantify the recovery performance of different methods, defined as:

24 $$RE=\frac{\mathop{min}\limits_{\phi \in \mathrm{[0,2}\pi )}||{e}^{-j\phi }{\bf{S}}-{{\bf{S}}}_{t}{||}^{2}}{||{{\bf{S}}}_{t}{||}^{2}},$$

where **S**_*t*_ is the true sample spectrum, and **S** denotes the reconstructed spectrum.

In the simulation experiments, we model a microscope setup realistically with its parameters as follows: the wavelength of incident light is 630 *nm*; CCD pixel size is 1.845 *μm*; the NA of the objective lens is 0.1. We use a 15 × 15 LED matrix as the light source to provide angle-varied illuminations. The distance between adjacent LED elements is 4 *mm*, and the distance between the sample and LED matrix is 90.88 *mm*. Besides, we use the ‘cameraman’ from ‘standard’ test images^28^ as the HR amplitude, and the ‘Aerial’ image (512 × 512 pixels) from the USC-SIPI image database^29^ is used as phase image. Using the Fourier ptychographic imaging formation, we simulated the ideal data with three sequential operations: (1) select different sub-regions of the HR Fourier domain caused by different incident angles, (2) inverse Fourier transform to the sub-region and get the LR plural image, (3) only retain the intensity of LR plural to obtain the ideal data. In addition, we also consider three different types of noises explicitly as follows:

- Gaussian noise: we added Gaussian white noise to the ideal data.
- Poisson noise: the ideal data is corrupted by Poisson-distributed noise at each pixel.
- Mixed Poisson-Gaussian noise: First, we simulated the data with Poisson noise. Then we added Gaussian white noise to the data corrupted with Poisson noise.

For Gaussian noise and the Gaussian component of the mixed Poisson-Gaussian, the standard deviation is the ratio between actual standard deviation and the maximum of the ideal data. Then we compared FPSR with three state-of-the-art methods, i.e., Newton method, TPWFP and GATFP. Besides, we also provide the result generated by NSR to show whether the benefit of the proposed method come form the noisy observing modeling or the sparse representation. The code of Newton method is adapted according to the code samples from http://sites.bu.edu/tianlab/open-source/. The code of TPWFP could be obtained at http://www.sites.google.com/site/lihengbian. In TPWFP, *α* ^*h*^ is set to 25, since it works well for the FPM reconstruction as stated in ref. 4. Another important parameter for all the algorithms is the iteration number. For TPWFP, GATFP, NSR and FPSR, 300 iterations are enough. We set 500 iterations for Newton method.

### Simulation results

First, we compare FPSR with the above mentioned three state-of-the-art methods and NSR to show their pros and cons. We apply each algorithm on the simulated data with Poisson noise, Gaussian noise and Poisson-Gaussian noise, respectively. The Poisson noise is used to describe the statistics of the incoming photons at each pixel, which is a discrete probability distribution^3^. The Gaussian noise is mostly caused in the capturing chain, such as thermal noise. Thermal noise is associated with the rapid and random motion of electrons within a conductor due to thermal agitation. Because the number of electrons in a conductor is very large, and their random motions are statistically independent, the central limit theorem indicates that thermal noise is Gaussian distributed with zero mean. A more realistic way to model the noise is a mixed Poisson-Gaussian distribution. Note that the standard deviation (std) for the Poisson-Gaussian noise denotes the level of the Gaussian component. The std of the Gaussian noise (Gaussian component of the mixed Poisson-Gaussian noise) is set to 2*e* − 3. And the ground truth noise variance is used in the synthetic experiment.

**Figure 1:**
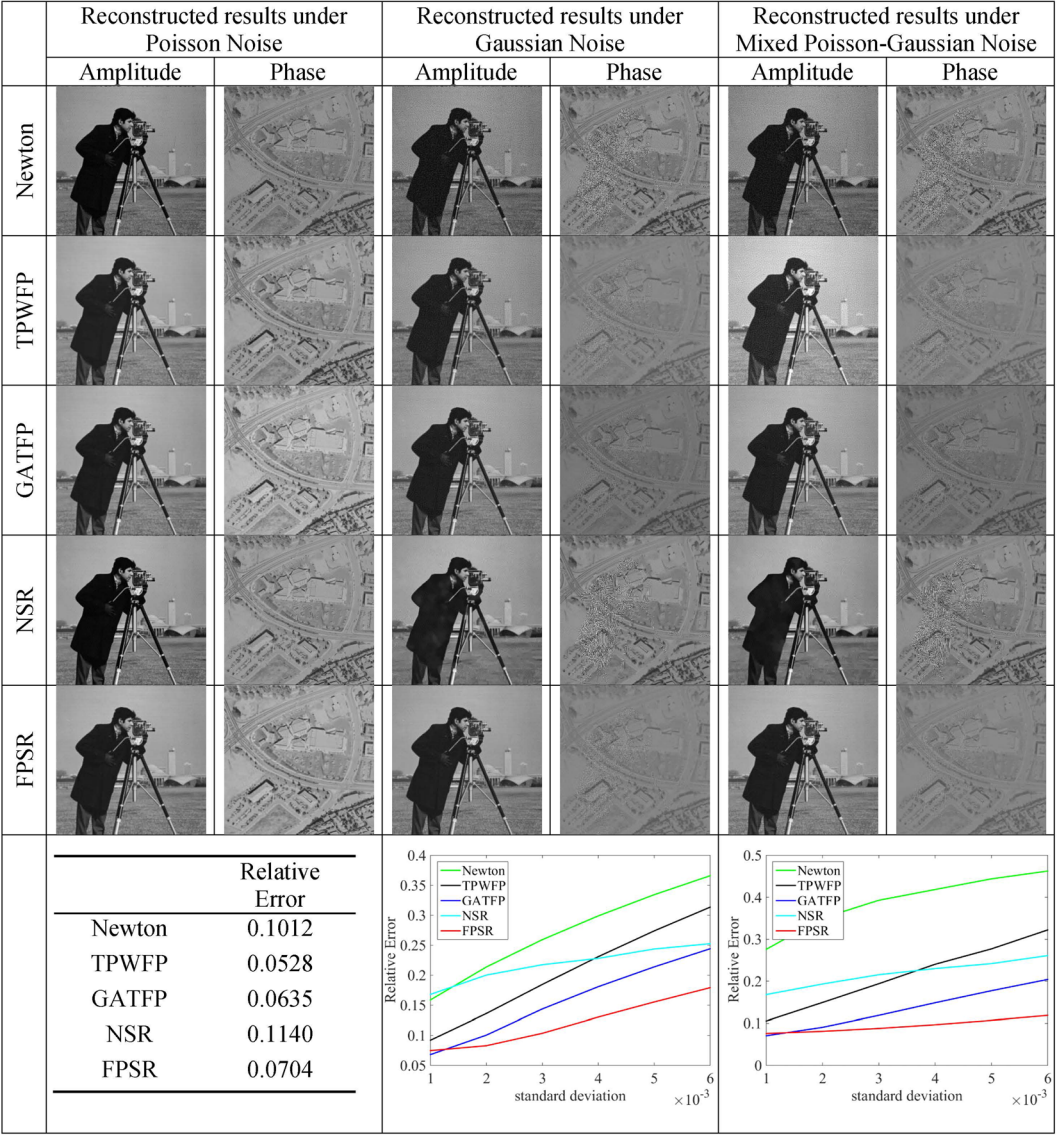
Reconstruction results with three types of noises (Poisson noise, Gaussian noise and mixed Poisson-Gaussian noise), using different algorithms (Newton method, TPWFP, GATFP, NSR and FPSR). This figure is not covered by the CC BY licence. Credits to copyright-holder of "cameraman" image: the Massachusetts Institute of Technology. All rights reserved, used with permission.

From the results (in Fig. 1), we can see that TPWFP performs well under Poisson noise, which benefits from its accurate Poisson signal model. Instead, Newton method and NSR minimizes the square of the difference between the actual and estimated measurements, which is the equivalent form of Gaussian likelihood function when the constant term is ignored^3^. Although NSR also incorporates the sparse representation, the performance of NSR is not as good as excepted. GATFP and FPSR can achieve a successful reconstruction without affecting the reconstruction quality. For Gaussian noise, GATFP and FPSR outperform the other three methods. This is because we also consider the Gaussian noise of the measurement explicitly as Eq. (5) in the noisy observation model. FPSR is advantageous than GATFP, since the sparse representaion could effectively reduce the noise by omitting the measurement under the certain threshold. TPWFP could recognize the most of Gaussian noise and remove them using the truncated Wirtinger gradient. However, for situations with high noise level, TPWFP may not be an effective reconstruction approach. Instead, Newton method estimates the background for each image and subtracts it to produce the corrected intensity image^30^. Obviously, NSR outperforms TPWFP and Newton method when the standard variance is large, however the advantage is not obvious under the case of smaller standard variance. For mixed Poisson-Gaussin noise, NSR achieves better performance than TPWFP and Newton method when standard variance is larger, but it suffers a significant degree of crosstalk in the phase image and generates some white spots in the amplitude image. GATFP obtains successful reconstruction. This is greatly attributed to its Poisson-Gaussian assumption. FPSR performs better than all the competing methods. This is mainly attributed to the sparse representation and mixed Poisson-Gaussian assumption. To conclude, we can see that the type of noise strongly influences the quality of reconstructed image, while FPSR is more robust under different types of noise. This behavior is well explained by the fact that our model can be treated as a generalized model of these types of noise.

All the five algorithms are implemented using MATLAB R2014a and the computer with the processor Intel(R) Core(TM) i5-3470@ 3.20GHz and 64bit Windows10 system. The complexity of the algorithms is characterized by the running time of 100 iterations, which is provided in Table 1. Since both NSR and FPSR employ the sparse representation, which involves the operations of BM3D frames for both amplitude and phase images, the running time of NSR and FPSR is much higher than the other three methods. Although the computational complexity is higher, the proposed FPSR achieves much better quality than other competing methods (Newton method, TPWFP, GATFP and NSR) for almost all the reconstructed results under different types of noises. With the emerging of more and more powerful computers, the running time of FPSR can be reduced significantly. Further studies on reducing the computational complexity of the proposed FPSR are also needed.

**Table 1:** Comparison of running time between state-of-the-arts methods and the proposed FPSR.

	Newton method	TPWFP	GATFP	NSR	FPSR
Running time (s)	193	83	40	630	658

### Experimental results

In this sub-section, we demonstrate the performance of our method with experimental results. The parameters of the real FPM imaging system are the same as those in the simulation. We obtain the estimated standard deviation of noise based on Median of Absolute Deviation (MAD) technique. However, the standard deviation for the Gaussian component of mixed Poisson-Gaussian noise might not be exact since the noise also contains the Poisson component. So we adjust the standard deviation based on the value obtained by MAD. In the experiment, we employ the blood smear and USAF target as samples, and capture a sequence of 225 images for both samples.

**Figure 2:**
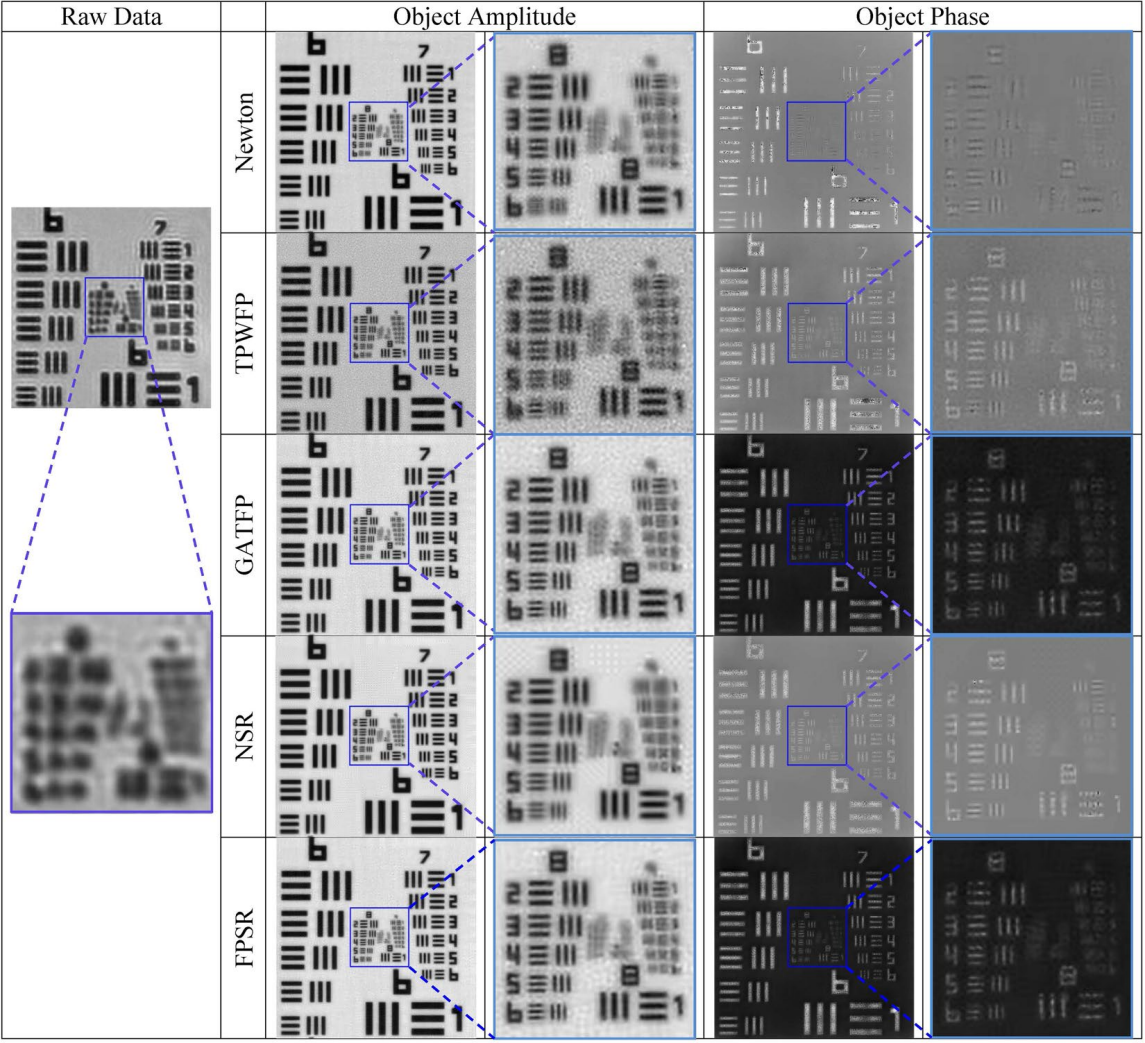
Reconstruction results over USAF target using different algorithms (Newton method, TPWFP, GATFP, NSR and FPSR).

**Figure 3:**
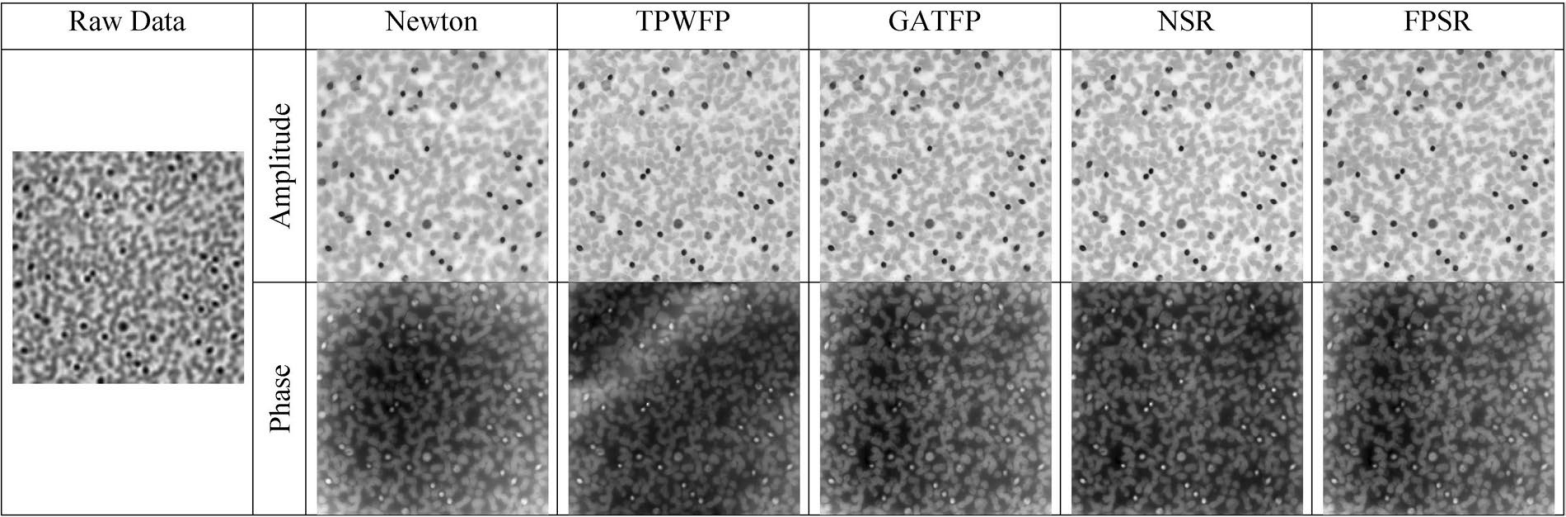
Reconstruction results over Blood smear using different algorithms (Newton method, TPWFP, GATFP, NSR and FPSR).

The reconstruction results over USAF target are shown in Fig. 2. In the result of Newton method, though most of the noise is removed due to the background subtraction step, many image details are subtracted as well (see the group 8 element 6). In the results generated by TPWFP, we can easily observe that TPWFP suffers noise corruption. The resluts of NSR suffer a significant degree of blurring (see the group 9 element 1). In addition, GATFP achieves good reconstruction with more details. Compared with GATFP, the reconstruction results using FPSR suffer from less noise in both amplitude and phase (see the part of zoom-in). The reconstruction results of blood smear are shown in Fig. 3. We can see that TPWFP and the Newton method produce fluctuations in the object phase over blood smear. GATFP, NSR and FPSR are able to remove the fluctuations in the object phase and achieve superior performance than the other competing methods. In addition, the reconstruction of the proposed FPSR has stronger contrast and contains less noises compared with GATFP and NSR. This is consistent with the simulation experiment. In all, FPSR offers a novel way for FPM to reconstruct highly accurate results suffered from noise-deteriorated inputs.

## Discussion

In this paper, we develop and test a novel reconstruction method for FPM termed as FPSR. By fully exploring the sparse priority of captured images, the proposed FPSR is formulated as a regularized optimization problem, which is solved by the Nash equilibrium algorithm. In FPSR, the data fidelity is constructed as a maximum likelihood problem, and the regulation term is expressed as a small number of nonzero elements over an appropriate basis for both amplitude and phase image. We compare the reconstruction quality of the proposed method and the competing methods under different types of noises. Both simulation and experimental results demonstrate the validity of our method.

One extension of FPSR is to handle much more complex noise by modeling data noise as a mixture of Gaussians. The mixture of Gaussian is a universal approximator to distributions and is able to fit a wide range of noises. In addition, we can also introduce more accurate approach for the estimation of standard deviation to improve the convergence speed and effectiveness of the algorithm.

The limitation of our method is the case that the model is non-convex, which might converge to incorrect local minima. In addition, GAT is able to stabilize the noise variance, yet the tails of variance stabilized coefficients distribution are empirically longer than normality as evidenced in ref. 31. Besides, when the noise is samll, FPSR may remove some useful information. Therefore, incorporating a convex program on FPSR to obtain a solution with minimum cost will be a research emphasis in the near future.
